# Applications of Nano-biofuel cells for Reiner-Philippoff nanoparticles with higher order slip effects

**DOI:** 10.1038/s41598-024-58476-y

**Published:** 2024-04-08

**Authors:** Abdulmajeed D. Aldabesh, Iskander Tlili

**Affiliations:** 1 Department of Mechanical Engineering, Faculty of Engineering, Al-Baha University, 65527 Al Bahah, Saudi Arabia; 2 Department of Physics, College of Science, Al-Zulfi, Majmaah University, 11952 Al-Majmaah, Saudi Arabia

**Keywords:** Nano-biofuel cells, Reiner–Philippoff nanofluid, Microorganisms, Thermal radiation, Porous medium, Numerical computations, Biomaterials, Nanoscale materials, Applied mathematics

## Abstract

Owing to advanced thermal features and stable properties, scientists have presented many novel applications of nanomaterials in the energy sectors, heat control devices, cooling phenomenon and many biomedical applications. The suspension between nanomaterials with microorganisms is important in biotechnology and food sciences. With such motivations, the aim of current research is to examine the bioconvective thermal phenomenon due to Reiner–Philippoff nanofluid under the consideration of multiple slip effects. The assessment of heat transfer is further predicted with temperature dependent thermal conductivity. The radiative phenomenon and chemical reaction is also incorporated. The stretched surface with permeability of porous space is assumed to be source of flow. With defined flow constraints, the mathematical model is developed. For solution methodology, the numerical simulations are worked out via shooting technique. The physical aspects of parameters are discussed. It is claimed that suggested results claim applications in the petroleum sciences, thermal systems, heat transfer devices etc. It has been claimed that the velocity profile increases due to Bingham parameter and Philippoff constant. Lower heat and mass transfer impact is observed due to Philippoff parameter.

## Introduction

The nanomaterials are the familiar class of fluids which are widely discussed in the thermal engineering and nanotechnology. With boosted and impressive thermal activities, investigators are focusing more novel applications of nanoparticles in the industrial, engineering and energy sectors. Basically, the nanofluids are decomposed materials of metallic particles with some base liquid. With low size and diameter, the properties of nanoparticles effectively boosted the thermal phenomenon. In nuclear system, cooling devices, solar energy, automated operation and many other processes, the key contributions of nanofluids have been reported. Choi^1^ presented a pioneer model for nanofluid by discussing some interesting properties. Later on, a wide contribution of researcher has been noted on nanomaterials with assumptions of different thermal sources. Gowda et al.^2^ suggested the Stefan blowing analysis in nanofluid flow with magnetic dipole features. Hayat et al.^3^ inspected the optimized onset in nanomaterial flow under induced induction of magnetic field. Amjad et al.^4^ focused towards the nanofluid applications in curved surface with Lorentz force. The Carreau nanofluid for radiative nanofluid flow via numerical treatment was conducted by Imran et al.^5^. Mabood et al.^6^ enrolled the additional slip features to observe the thermal treatment of nanofluid. Xu et al.^7^ prepared the thermal nanofluid model with third grade base fluid to boost the heat transfer pattern. Chen et al.^8^ examined the thermally developed analysis for ternary nanoparticles via modified Fourier’s approach. Arif et al.^9^ discussed the couple stress-Casson nanofluid flow with fractional assessment. Analysis for Sutterby nanofluid via stretched cylinder was explored by Abbas et al.^10^. Khan et al.^11^ analyzed the water-graphene thermal performance in shrinking surface with additional impact of chemical reaction. Vadiya et al.^12^ reflected the suspended thermal results for nanoparticles with Phan–Thien–Tanner model. The analysis was reported due to channel flow. Maatoug et al.^13^ claimed the hydraulic systems applications for nanofluid via lubricated surface. Ali et al.^14^ executed heat transfer observations for iron doped zinc-oxide nanoparticles following the bulk motion. The rotating flow due to stretchable disk with nanofluid interaction was examined by Qayyum et al.^15^. Tariq et al.^16^ determined the improvement in engine oil thermal performances due to graphene nanoparticles. Irfan et al.^17^ observed the fluctuation in heat aspects due to Carreau nanofluid flow with Joule heating applications. The energy transport of Maxwell nanofluid with thermos-diffusion contribution was inspected by Irfan et al.^18^. Irfan^19^ executed the thermophoretic analysis for nanofluid comprising the Joule heating impact. Anwar et al.^20^ examined the clay based nanomaterials thermal efficiencies with Newtonian heating phenomenon. Hamid et al.^21^ determined the inspection of heat transfer due to square shaped cavity with heated and cold block.

The bioconvection phenomenon is an interesting pattern of microorganisms which float with lower densities. Due to distinct instability frame, the microorganisms swim in upper portion of fluid which make it denser. Such denser effects are due to peak stratification density. The interaction in nanomaterials amongst the microorganisms is important due to stability point of view. The bioconvection applications are commonly examined in the biofuels, fertilizers and food industry. Hosseinzadeh et al.^22^ analuzed the suspension of microorganisms for cross fluid in 3-D flow. Tlili et al.^23^ discussed the partial slip impact for bioconvection phenomenon of micropolar nanofluid. Iqbal et al.^24^ executed bioconvective thermal analysis for Riga supported flow containing water base liquid. Bafakeeh et al.^25^ analyzed the viscoelastic nanofluid with microorganisms decomposition containing variable thermal conductivity. Khan et al.^26^ discussed the bioconvection phenomenon in disk flow. The nonlinearly thermally supported bioconvection flow of nanoparticles was predicted by Bhatti et al.^27^.

This research aims to present the bioconvective phenomenon due to Reiner–Philippoff nanofluid model subject to various thermal sources. The thermal model is developed in view of following novel features:

- A two-dimensional steady transport of Reiner–Philippoff nanofluid with decomposition of microorganisms have been investigated due to porous stretched surface.
- The thermal properties of Reiner–Philippoff nanofluid are examined with variable thermal conductivity assessment.
- Multiple slip features are utilized for entertaining the thermal analysis.
- The linear thermal radiation effects are endorsed to the model which are further proceeded via effective Prandtl approach.
- The extension in concentration equation is contributed with chemical reaction features.
- After developing the model, numerical treatment is done via shooting algorithm.
- A detailed physical aspect of parameters with different engineering applications have been studied.

It is further emphasized that although different analysis is available on bioconvection flow of nanoparticles, however, no such contribution is performed for Reiner–Philippoff nanofluid with variable thermal conductivity and higher order slip effects. The rheology of Reiner–Philippoff fluid model is interesting and dynamical which is discussed in current continuation. The fluid flow is subject to the stretching surface. The phenomenon of stretching surface flows is commonly observed in the manufacturing processes like crystal growing, fiber production, extrusion of metals, wire drawing, polymers etc.

## Formulation of problem

A steady two-dimensional transport of Reiner–Philippoff nanofluid is assumed confined by a uniform motion of moving surface. The nanofluid convey the applications of bioconvection phenomenon due to suspension of microorganisms. The magnetic force is utilized via normal approach. The porous medium effects are assumed due to permeability of porous space. The nanofluid convey temperature dependent thermal conductivity. For 2-D flow, the velocity component $u$ is taken along the surface while normal surface velocity component is expressed with $v$. Expressing temperature, concentration and microorganisms density $T,c$ and $n$, respectively. The radiative effects are utilized. The expression for Reiner–Philippoff via stress tensor is presented as^28–30^:

1 $$\frac{\partial u}{{\partial y}} = \frac{\tau }{{\mu_{\infty } + \frac{{\mu_{0} - \mu_{\infty } }}{{1 + \left( {\frac{\tau }{{\tau_{S} }}} \right)}}}},$$

with shear stress $\tau$ and rate of deformation $\frac{\partial u}{{\partial y}}.$

Based on these constraints, the governing modelled equations are:

2 $$\frac{\partial u}{{\partial x}} + \frac{\partial u}{{\partial y}} = 0,$$

3 $$u\frac{\partial u}{{\partial x}} + v\frac{\partial u}{{\partial y}} = \frac{1}{{\rho_{f} }}\frac{\partial \tau }{{\partial y}} - \frac{{\sigma_{ * } B_{0}^{2} }}{{\rho_{f} }}u - \frac{\nu \Phi }{{k_{s} }}u,$$

4 $$u\frac{\partial T}{{\partial x}} + v\frac{\partial T}{{\partial y}} = \frac{1}{{\left( {\rho c} \right)_{f} }}\frac{\partial }{\partial y}K\left( T \right)\left( {\frac{\partial T}{{\partial y}}} \right) + \frac{{16T_{\infty }^{3} \sigma_{m} }}{{3\left( {\rho c} \right)_{f} k_{m} }}\frac{{\partial^{2} T}}{{\partial y^{2} }} + \tau_{f} \left[ {D_{T} \frac{\partial C}{{\partial y}}\frac{\partial T}{{\partial y}} + \frac{{D_{T} }}{{T_{\infty } }}\left( {\frac{\partial T}{{\partial y}}} \right)^{2} } \right],$$

5 $$u\frac{\partial c}{{\partial x}} + v\frac{\partial c}{{\partial y}} = D_{B} \frac{{\partial^{2} c}}{{\partial y^{2} }} + \frac{{D_{T} }}{{T_{\infty } }}\frac{{\partial^{2} T}}{{\partial y^{2} }} - Kr\left( {c - c_{\infty } } \right),$$

6 $$u\frac{\partial n}{{\partial x}} + v\frac{\partial n}{{\partial y}} + \frac{bw}{{\left( {c_{w} - c_{\infty } } \right)}}\left[ {\frac{\partial }{\partial y}\left( {n\frac{\partial c}{{\partial y}}} \right)} \right] = D_{m} \frac{{\partial^{2} n}}{{\partial y^{2} }},$$

The novel physical quantities are fluid viscosity $\rho_{f}$, electric conductivity $\left( {\sigma_{ * } } \right),$ permeability of porous space $k_{s}$, porous medium $\Phi$, variable thermal conductivity $K\left( T \right)$, thermal capacity of nanoparticles to fluid ratio $\tau_{f}$, Brownian coefficient $D_{B}$, thermophoresis coefficient $D_{T}$, Stefan-Boltzmann constant $\sigma_{m}$, mean absorption coefficient $k_{m}$, swimming cells speed $w$, chemotaxis constant $b$, microorganisms diffusion constant $D_{m}$.

In Eq. (4), defining the variable thermal conductivity $K\left( T \right)$ is:

7 $$K\left( T \right) = K_{\infty } \left( {1 + \alpha \frac{{T - T_{\infty } }}{\Delta T}} \right),$$

With variable thermal conductivity coefficient $\alpha$.

### Boundary constraints

The developed model is based on following boundary conditions^6^:

8 $$\begin{aligned} u & = u_{w} \left( x \right) = ax^{\frac{1}{3}} + u_{slip} ,\,u_{slip} = A\frac{\partial u}{{\partial y}} + B\frac{{\partial^{2} u}}{{\partial y^{2} }},\,\,v = 0, \\ & \quad - k\frac{\partial T}{{\partial y}} = h_{f} \left( {T_{f} - T} \right),\,\,c = c_{w} ,\,\,n = n_{w} ,\quad at\;y = 0, \\ \end{aligned}$$

9 $$u \to 0,T \to T_{\infty } ,c \to c_{\infty } ,n \to n_{\infty } \quad as\quad y \to \infty .$$

First boundary constraint is associated to the slip flow phenomenon with slip coefficients $A$ and $B$. For heat transfer, convective boundary conditions are implemented with thermal conductivity $k$ and heat transfer coefficient $h_{f}$.

### Dimensionless variables

The system of partial differential Eqs. (2–6) is converted into ordinary differential equations by introducing following dimensionless variables^30^:

10 $$\begin{aligned} & \eta = \sqrt {\frac{a}{\nu }} \frac{y}{{x^{\frac{1}{3}} }},\psi = \sqrt {a\nu } x^{\frac{2}{3}} ,\;\tau = \sqrt {a^{3} \nu } g\left( \eta \right),\;\theta \left( \eta \right) = \frac{{T - T_{w} }}{{T_{w} - T_{\infty } }}, \\ & \phi \left( \eta \right) = \frac{{c - c_{w} }}{{c_{w} - c_{\infty } }},\;\chi \left( \eta \right) = \frac{{n - n_{w} }}{{n_{w} - n_{\infty } }}. \\ \end{aligned}$$

The dimensionless system is:

11 $$g = f^{\prime\prime}\frac{{g^{2} + \lambda S^{2} }}{{g^{2} + S^{2} }},$$

12 $$g^{\prime} = \frac{1}{3}f^{{\prime}{2}} - \frac{2}{3}ff^{\prime\prime} - Hf^{\prime} - \beta f^{\prime} = 0$$

13 $$\left( {\frac{1 + Rd}{{\Pr }}} \right)\left[ {\left( {1 + \alpha \theta } \right)\theta ^{\prime\prime} + \alpha \left( {\theta ^{\prime}} \right)^{2} } \right] + f\theta ^{\prime} + Nb\theta ^{\prime}\phi ^{\prime} + Nt\left( {\theta ^{\prime}} \right)^{2} = 0,$$

14 $$\phi^{\prime\prime} + Scf\phi^{\prime} + \frac{Nt}{{Nb}}\theta^{\prime\prime} - Sc\delta \phi = 0$$

15 $$\chi^{\prime\prime} + Lbf\chi^{\prime} - Pe\left[ {\phi^{\prime\prime}\left( {\chi + \delta_{m} } \right) + \chi^{\prime}\phi^{\prime}} \right] = 0,$$

16 $$\begin{aligned} & f\left( 0 \right) = 0,f^{\prime}\left( 0 \right) = 1 + \omega_{1} f^{\prime\prime}\left( 0 \right) + \omega_{2} f^{\prime\prime\prime}\left( 0 \right)\,,\theta^{\prime}\left( 0 \right) = - Bi\left( {1 - \theta \left( 0 \right)} \right), \\ & \phi \left( 0 \right) = 1,\,\,\chi \left( 0 \right) = 1 \\ & f^{\prime}\left( \infty \right) \to 0,\theta \left( \infty \right) \to 0,\phi \left( \infty \right) \to 0,\chi \left( \infty \right) \to 0. \\ \end{aligned}$$

In current analysis, the radiative phenomenon is discussed via effective Prandtl number approach. Modifying Eq. (13) as:

17 $$\left[ {\left( {1 + \alpha \theta } \right)\theta ^{\prime\prime} + \alpha \left( {\theta ^{\prime}} \right)^{2} } \right] + \Pr_{eff} \left[ {f\theta ^{\prime} + Nb\theta ^{\prime}\phi ^{\prime} + Nt\left( {\theta ^{\prime}} \right)^{2} } \right] = 0,$$

where $\Pr_{eff} = \Pr /\left( {1 + Rd} \right)$ is the effective Prandtl number.

The dimensionless variables are Philippoff fluid parameter $\lambda$, Bingham number $S$, Hartmann number $H$, porosity constant $\beta$, Brownian constant $Nb$, thermophoresis constant $Nt$, chemical reaction parameter $\delta = \frac{Kr}{a}$, Schmidt number $Sc$, slip factors $\omega_{1} ,\omega_{2}$, bioconvection Lewis number $Lb$, microorganism difference parameter $\delta_{m}$ Peclet number $Pe$ and Biot number $Bi$ with following definitions:

$$\begin{aligned} \lambda & = \frac{{\mu_{0} }}{{\mu_{\infty } }},\;S = \frac{{\tau_{S} }}{{\sqrt {a^{3} \nu } }},\;H = \frac{{\sigma_{ * } B_{0}^{2} }}{{a\rho_{f} }},\;\beta = \frac{\nu \Phi }{{ak_{s} }},\;Nb = \tau_{f} D_{B} \left( {c_{w} - c_{\infty } } \right)/\nu ,\;Nt = \tau_{f} D_{T} \left( {T_{w} - T_{\infty } } \right)/T_{\infty } \nu \\ \delta & = \frac{Kr}{a},\;Sc = \frac{\nu }{{D_{B} }},\;\omega_{1} = A\sqrt {\frac{a}{\nu }} ,\omega_{2} = B\sqrt {\frac{a}{\nu }} ,\;Lb = \frac{\nu }{{D_{m} }},\;\delta_{m} = \frac{{N_{\infty } }}{{N_{w} - N_{\infty } }},\;Pe = \frac{bw}{{D_{m} }},\;Bi = \frac{{h_{f} }}{k}\sqrt {\frac{\nu }{a}} . \\ \end{aligned}$$

### Physical quantities of interest

The effective Nusselt number $Nu_{x}^{ * }$, Sherwood number $Sh_{x}$ and motile density number $Nn_{x}$ are defined as:

18 $$\begin{aligned} & Sh_{x} \left( {{\text{Re}}_{x} } \right)^{ - 0.5} = - \phi ^{\prime}\left( 0 \right), \\ & Nu_{x}^{ * } \left( {{\text{Re}}_{x} } \right)^{ - 0.5} = - \theta ^{\prime}\left( 0 \right), \\ & Nn_{x} \left( {{\text{Re}}_{x} } \right)^{ - 0.5} = - \chi ^{\prime}\left( 0 \right). \\ \end{aligned}$$

## Numerical scheme

In order to presents the numerical evaluation of problem, the shooting technique has been implemented. The motivations for utilizing the shooting method is due to excellent convergence. An excellent accuracy of shooting technique has been observed. This scheme is based on conversion of boundary value problem into initial value system as follows:

19 $$\begin{aligned} f & = \Lambda_{1} ,f^{\prime} = \Lambda_{2} ,f^{\prime\prime} = \Lambda_{3} ,f^{\prime\prime\prime} = \Lambda^{\prime}_{3} , \\ \theta & = \Lambda_{4} ,\theta^{\prime} = \Lambda_{5} ,\theta^{\prime\prime} = \Lambda^{\prime}_{5} , \\ \phi & = \Lambda_{6} ,\phi^{\prime} = \Lambda_{7} ,\phi^{\prime\prime} = \Lambda^{\prime}_{7} , \\ \chi & = l_{8} ,\chi^{\prime} = l_{9} ,\chi^{\prime\prime} = l^{\prime}_{9} , \\ \end{aligned}$$

20 $$\begin{aligned} \Lambda^{\prime}_{3} & = \frac{1}{3}\Lambda_{2}^{2} - \frac{2}{3}\Lambda_{1} \Lambda_{2}^{2} - H\Lambda_{2} - \beta \Lambda_{2} , \\ \Lambda^{\prime}_{5} & = - \frac{{Pr_{eff} \left( {\Lambda_{1} \Lambda_{5} + Nt\Lambda_{5}^{2} + Nb\Lambda_{5} \Lambda_{7} } \right) - \alpha \Lambda_{5}^{2} }}{{1 + \alpha l_{4} }}, \\ \Lambda^{\prime}_{7} & = - Sc\Lambda_{1} \Lambda_{7} - \frac{Nt}{{Nb}}l^{\prime}_{5} + Sc\delta \Lambda_{6} , \\ \Lambda^{\prime}_{9} & = \Lambda_{7} \Lambda_{9} - Lb\Lambda_{1} \Lambda_{9} + Pe\left( {l_{8} + \varpi } \right)\Lambda^{\prime}_{7} . \\ \end{aligned}$$

with

21 $$\begin{aligned} & \Lambda_{1} \left( 0 \right) = 0,\,\,\,\Lambda_{2} \left( 0 \right) = 1 + \gamma_{1} \Lambda_{3} \left( 0 \right) + \gamma_{2} \Lambda^{\prime}_{3} \left( 0 \right)\,\,\,\,,q_{5} \left( 0 \right) = - Bi\left( {1 - \Lambda_{4} \left( 0 \right)} \right), \\ & \Lambda_{5} \left( 0 \right) = 1,\,\,\Lambda_{8} \left( 0 \right) = 1, \\ & \Lambda_{2} \left( \infty \right) \to 0,\,\,\,\,\Lambda_{4} \left( \infty \right) \to 0,\,\,\,\,\Lambda_{6} \left( \infty \right) \to 0,\,\,\,\Lambda_{8} \left( \infty \right) \to 0. \\ \end{aligned}$$

## Physical interpretation of results

Physical interpretation of results is presented in this section. The graphical analysis is subject to some fixed numerical values of flow parameters like $\lambda = 0.2,$ $S = 0.5,$ $H = 0.5,$ $\beta = 0.6,$ $Nb = 0.3,$ $Nt = 0.2,$ $\delta = 0.4,$ $Sc = 0.5,$ $\omega_{1} = \omega_{2} = 0.2$, $Lb = 0.3$ and $\delta_{m} = 0.5.$

### Velocity profile

**Figure 1:**
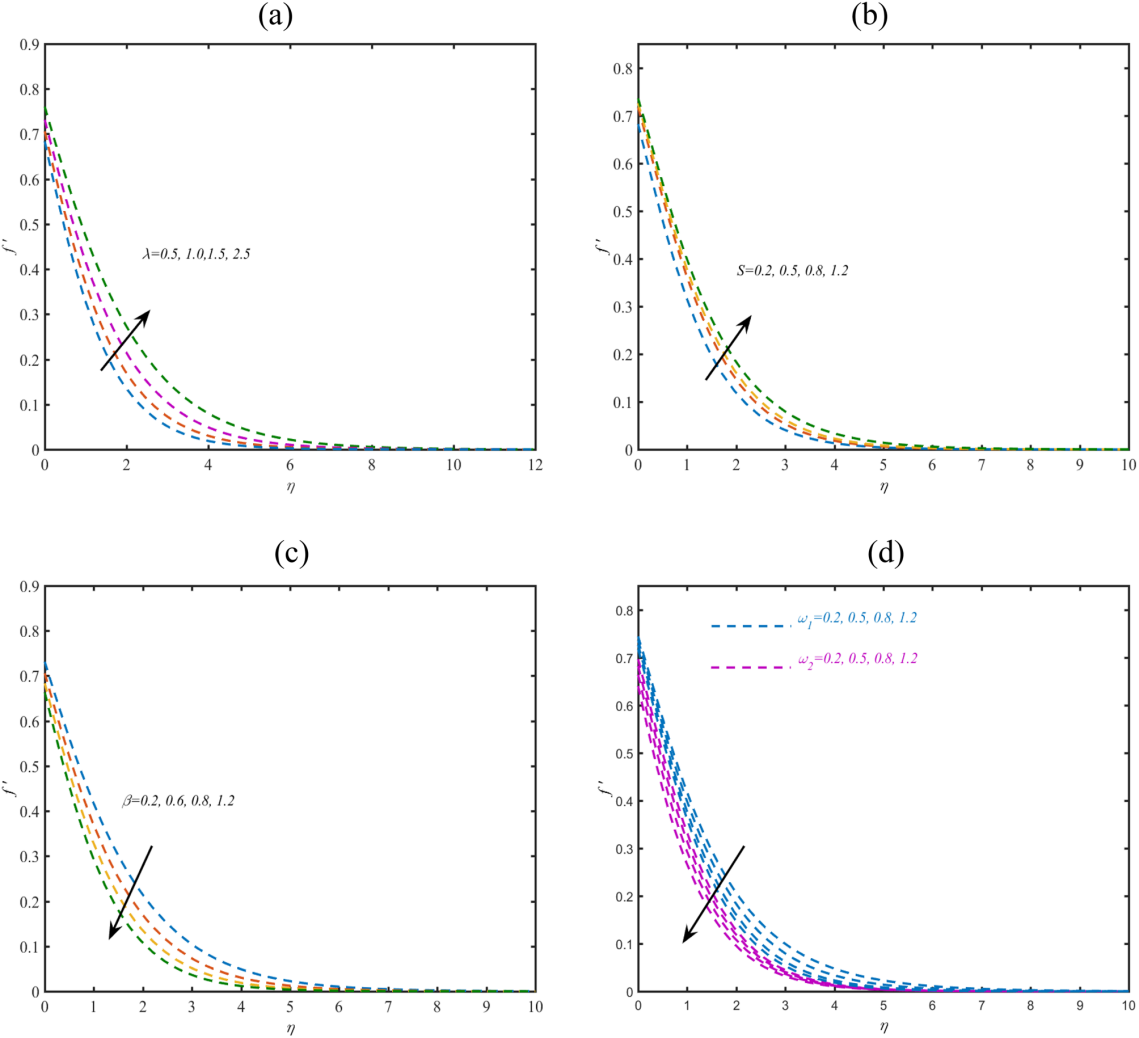
(**a**–**d**) Profile of $f^{\prime}$ with variation of (**a**) $\lambda$ (**b**) (**c**) $S$ (**d**) $\omega_{1} ,\omega_{2}$.

The truncated profile of velocity $f^{\prime}$ with assigning appropriate change to Philippoff constant $\lambda$ is incorporated via Fig. 1a. The increasing trend in variation of $f^{\prime}$ is noticed when $\lambda$ assigning maximum variation. Such increasing effects are due to distinct rheology and classical nature of Reiner-Philippoff model. Figure 1b shows the prospective of $f^{\prime}$ due to larger variation of Bingham number $S.$ Physically, Bingham number presents ratio between yield stress to viscous stress. Larger variation of $\lambda$ leads to less viscous stress which needs to be enhancement in velocity. The deduced results show an increment in $f^{\prime}$ due to $S.$ Physical onset of porosity constant $\beta$ on $f^{\prime}$ is depicted via Fig. 1c. Here, a control change in velocity is announced for larger $\beta$. Physically, the decrement in velocity due to porosity constant is associated to the permeability of porous space. Due to tiny pores, a loss in the fluid is noted which declined the velocity rate. Figure 1d disclosing the behavior of velocity due to interaction of slip parameters $\omega_{1}$ and $\omega_{2}$. The decreasing nature of velocity is proceeded due to both slip coefficients. The interaction of slip effectively reduces the velocity within the confine regime effectively.

### Temperature profile

**Figure 2:**
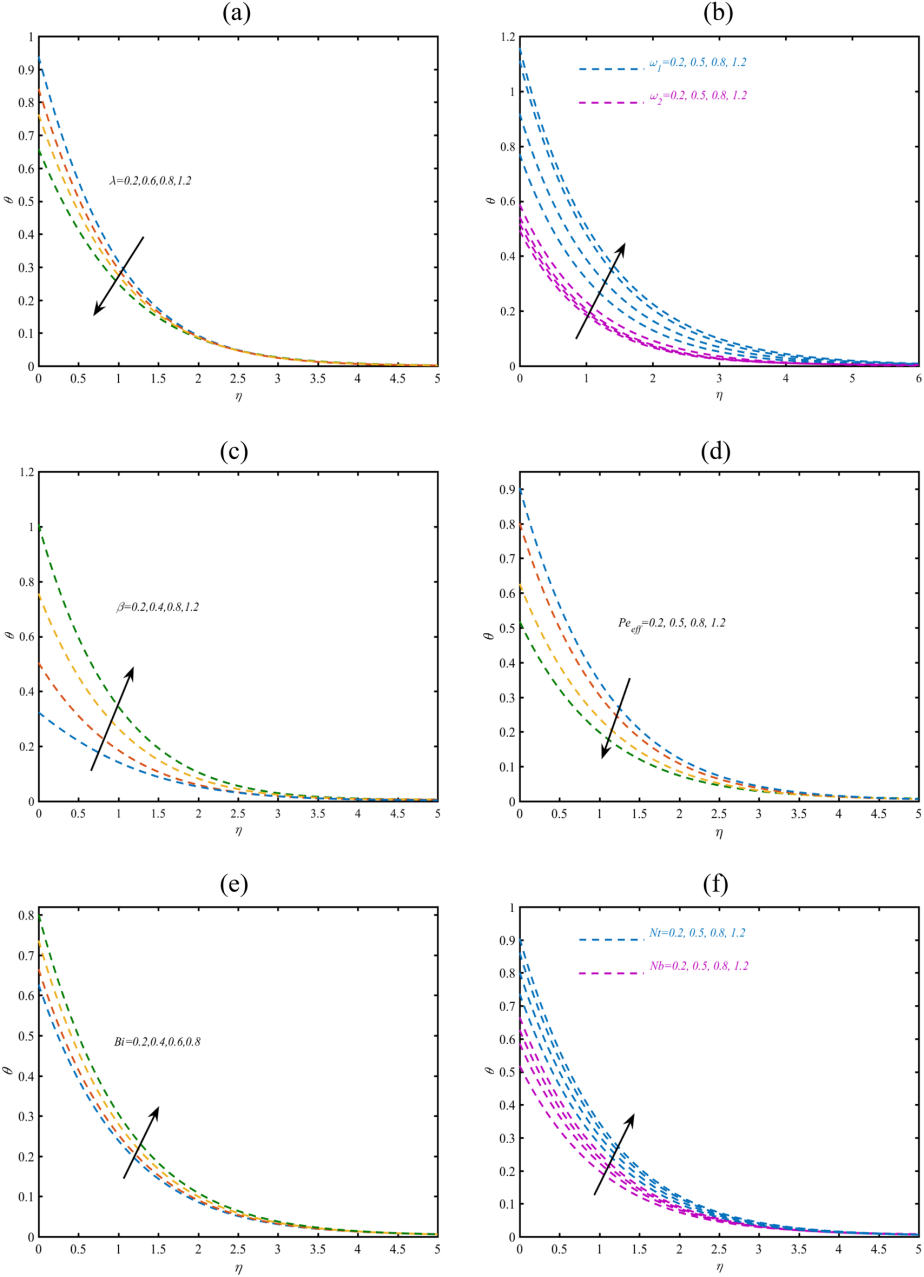
(**a**–**f**) Profile of $\theta$ with variation of (**a**) $\lambda$ (**b**) $\omega_{1} ,\omega_{2}$ (**c**) $\beta$ (**d**) $\Pr_{eff}$, (**e**) $Bi$ and (**f**) $Nb,Nt$.

Figure 2a presents the effects of Philippoff parameter $\lambda$ on temperature profile $\theta .$ The reduction in heat transmission is observed with varying $\lambda$. In Fig. 2b, the effective outcomes of $\omega_{1}$ and $\omega_{2}$ on $\theta$ has been studied. The presence of slip parameters enhances the heat transfer prediction. Therefore, presence of slip factors can boost the thermal process. Figure 2c claiming the influence of porosity constant $\beta$ on $\theta .$ The enhanced outcomes are listed via increasing $\beta$ due to permeability of porous space. These observations are important in petroleum industry and soil sciences. Figure 2d suggested the role of effective Prandtl number $\Pr_{eff}$ on $\theta .$ The effective Prandtl number is the combination of traditional Prandtl constant and thermal radiation parameter. Lower results for heat transfer rate is pronounced against $\Pr_{eff}$. The effective Prandtl constant present a direct relation with Prandtl number which means that thermal diffusivity also declined due to $\Pr_{eff}$. In order to assessing the prediction of thermal Biot number $Bi$ on $\theta$, Fig. 2e is prepared. The increasing role of $Bi$ are being noticed in profile of $\theta .$ Physically, the thermal Biot constant preserve a direct relation with coefficient of heat transfer due to which temperature boosted. Figure 2f suggested the contribution of thermophoresis parameter $Nt$ and Brownian constant $Nb$ on $\theta .$ As expected enhancement in is resulted for $Nt$ and $Nb$. The thermophoresis constant reflects the motion of migrated particles form relatively heated to cooler region. This migration of fluid particles turns to enhancement in temperature. The enhancing outcomes for $\theta$ in presence of $Nb$ are physically attributed due to random fluid movement. Moreover, it is also important to note that heat transfer rate boosted relatively more progressive due to thermophoresis constant.

### Concentration profile

**Figure 3:**
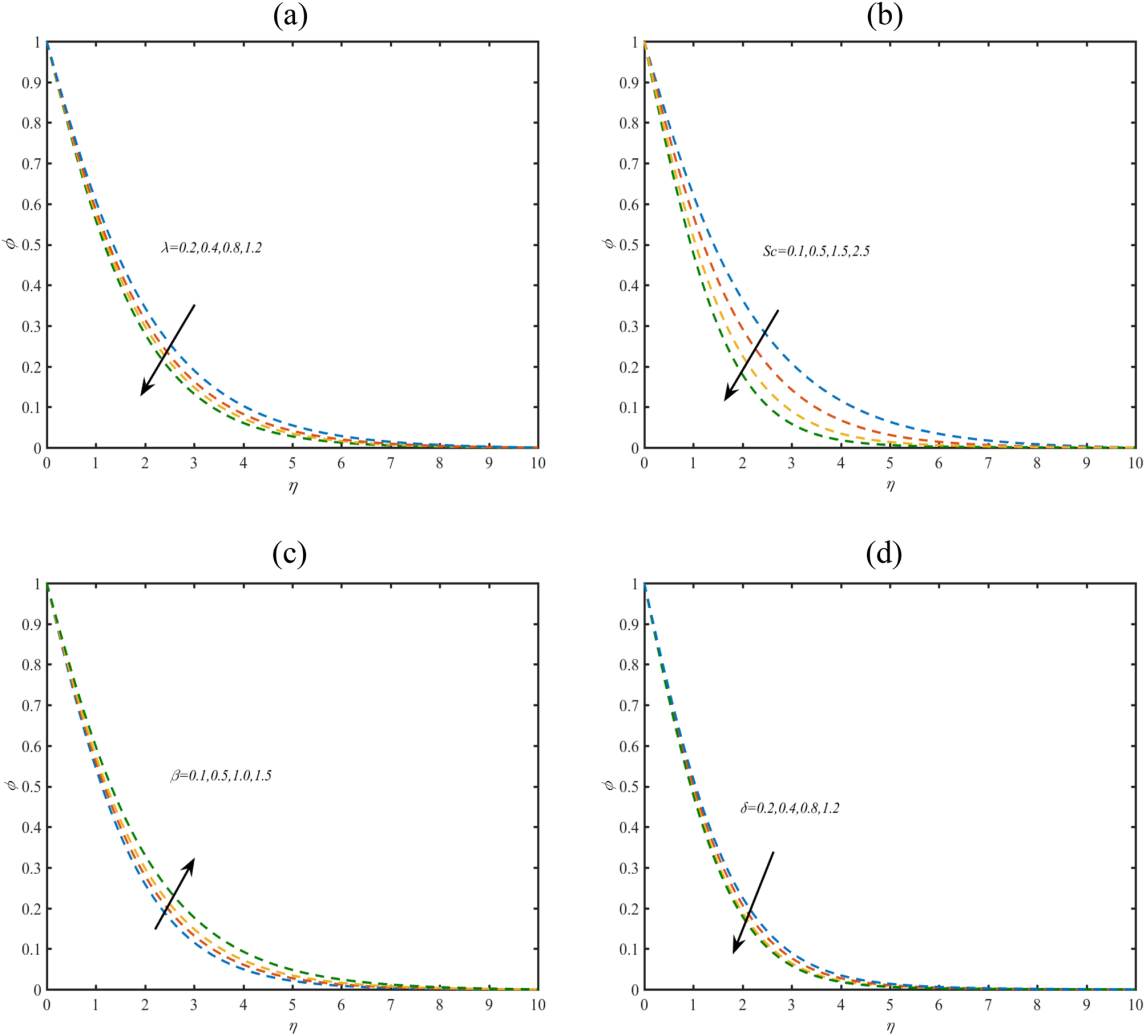
(**a**–**d**) Profile of $\phi$ with variation of (**a**) $\lambda$ (**b**) $Sc$ (**c**) $\beta$ and $\delta$.

Figure 3a reports the assessment of concentration field $\phi$ due to specified numerical values of Philippoff parameter $\lambda$. The concentration of suspended nanofluid reduces with $\lambda$. Figure 3b aims to judge the effects of Schmidt constant $Sc$ on $\phi$. With increasing $Sc$, the mass diffusivity reduces which turning down the concentration phenomenon. In order to interpreting the results for $\phi$ subject to porosity constant $\beta$, Fig. 3c is prepared. The increasing role of $\beta$ on $\phi$ has been predicted. Such outcomes are due to presence of permeability of porous space. Figure 3d shows that concentration is lower when chemical reaction parameter $\delta$ contributes.

### Microorganisms profile

**Figure 4:**
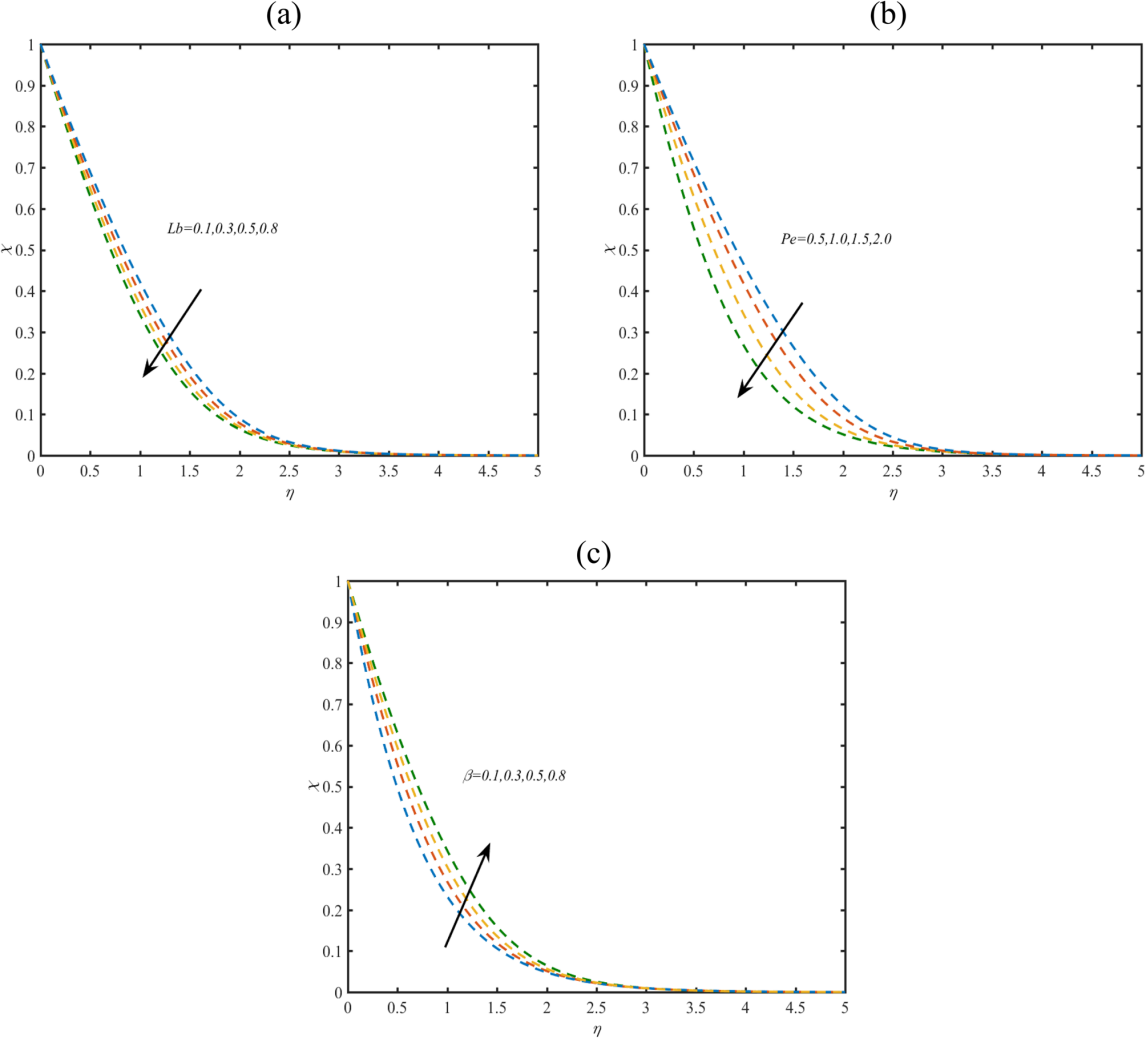
(**a**–**c**) Profile of $\chi$ with variation of (**a**) $Lb$ (**b**) $Pe$ (**c**) $\beta$.

Figure 4a aiming to reports the microorganism profile $\chi$ for bioconvected Lewis number $Lb$. The lower effects of $Lb$ on $\chi$ are noted. Figure 4b demonstrates the significance of Peclet number $Pe$ on $\chi$. The microorganisms field get reduces for $Pe$. The decrement in $\chi$ due to $Pe$ is resulted due to low microorganisms diffusivity. Figure 4c shows that $\chi$ is lower for $\lambda$.

### Streamlines

**Figure 5:**
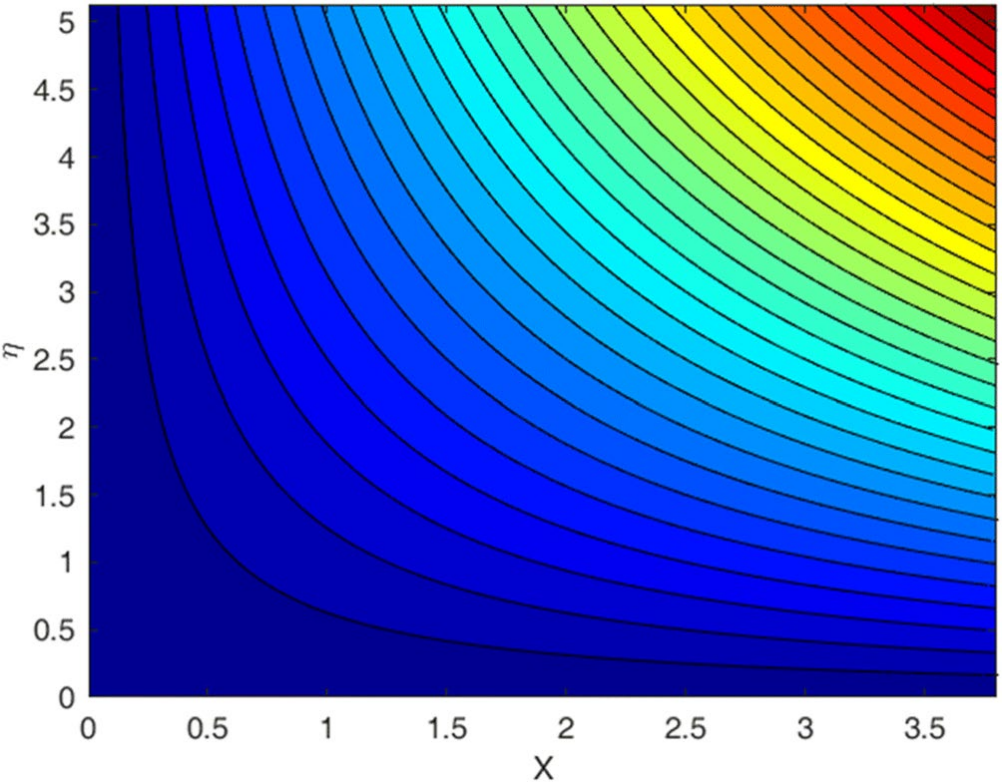
Streamlines for flow problem.

The flow pattern is observed by plotting streamlines in Fig. 5. A smooth flow behavior is observed for current slip flow problem.

### Physical quantities

Table 1 presents the numerical outcomes for $- f^{\prime\prime}\left( 0 \right)$ due to variation of $\lambda$, $H$ and $\beta$. Increasing effects of $H$ and $\beta$ on $- f^{\prime\prime}\left( 0 \right)$ are noted. However, $- f^{\prime\prime}\left( 0 \right)$ reduces due to $\lambda$. Table 2 reports the variation of parameters on $- \theta^{\prime}\left( 0 \right), - \phi^{\prime}\left( 0 \right)$ and $- \chi^{\prime}\left( 0 \right)$. With enhancing $S$ and $\lambda$, these quantities result lower variation. However, larger effects are noted due to $\Pr_{eff}$. Furthermore, interaction of slip parameters also presents reduction in these quantities.

**Table 1:** Numerical values of $- f^{\prime\prime}\left( 0 \right)$ for different parameter.

$\lambda$	$\beta$	$H$	$- f^{\prime\prime}\left( 0 \right)$
0.1	0.2	0.4	0.42414369
0.3			0.40324543
0.5			0.37321434
0.9			0.35074356
0.2	0.3		0.44434316
	0.5		0.46354145
	0.7		0.48764853
	0.9		0.51245456
		0.2	0.41804621
		0.6	0.43045254
		0.8	0.46535655
		1.2	0.48433424

**Table 2:** Numerical values of $- \theta^{\prime}\left( 0 \right), - \phi^{\prime}\left( 0 \right)$ and $- \chi^{\prime}\left( 0 \right)$ for different parameter.

$\lambda$	$\Pr_{eff}$	$S$	$\omega_{1}$	$\omega_{2}$	$- \theta^{\prime}\left( 0 \right)$	$- \phi^{\prime}\left( 0 \right)$	$\chi^{\prime}\left( 0 \right)$
0.1	0.5	0.2	0.3	0.3	0.526774	0.436265	0.436886
0.3					0.492462	0.388046	0.426433
0.5					0.466784	0.346478	0.395966
0.7					0.433567	0.3156753	0.376557
0.2	0.2				0.5434454	0.4997889	0.417674
	0.4				0.5953763	0.526537	0.436488
	0.6				0.626784	0.546537	0.46657
	0.8				0.654356	0.5734526	0.4954643
		0.3			0.47436	0.3876543	0.3321662
		0.5			0.4554762	0.3514527	0.317294
		0.9			0.432879	0.3423457	0.298678
		1.2			0.385764	0.310768	0.271435
			0.2		0.452333	0.381846	0.339785
			0.4		0.430465	0.374625	0.317644
			0.6		0.416573	0.356478	0.291256
			0.8		0.386784	0.323451	0.260543
			0.8	0.2	0.517565	0.434874	0.333425
				0.6	0.491155	0.405353	0.317986
				1.0	0.460643	0.385242	0.287547
				1.2	0.436365	0.367358	0.265334

## Conclusions

A bioconvective-thermal model for radiative flow of Reiner–Philippoff nanofluid with significance of multiple slip have been studied. The assessment in thermal phenomenon is predicted under the variable thermal conductivity. The numerical simulations are performed for modeled problem. Some interesting observations are:

- The velocity profile is increasing for Philippoff constant and Bingham parameter.
- The presence of multiple slip reduces the interaction velocity.
- With enhancing effective Prandtl number and Philippoff constant, the temperature profile reduces.
- Increasing outcomes are noted for temperature due to variation of porosity parameter and slip coefficients.
- The concentration profile reduces with chemical reaction constant and Philippoff constant.
- The wall shear force declined with Philippoff constant.
- Nusselt number, motile density number and Sherwood number reduces with slip parameters.
